# MALDI-TOF MS protein fingerprinting of mixed samples

**DOI:** 10.1093/biomethods/bpz013

**Published:** 2019-09-25

**Authors:** Michael A Reeve, Denise Bachmann

**Affiliations:** Department of Bioscience, CABI Bioscience, Bakeham Lane, Egham, Surrey TW20 9TY, UK

**Keywords:** matrix-assisted laser-desorption and ionization time-of-flight mass spectrometry, mixtures of microorganisms, rice-associated bacteria, spectral complexity, spectral comparison algorithms

## Abstract

Analytical techniques currently available for the characterization of mixtures of microorganisms are generally based on next-generation sequencing. Motivated to develop practical and less-expensive methods for characterizing such mixtures, we propose, as an alternative or complement, the use of matrix-assisted laser-desorption and ionization time-of-flight mass spectrometry (MALDI-TOF MS), which is capable of high-resolution discrimination between species and even between biotypes within species. Potential approaches employing this technique for such characterization are discussed along with impediments to their successful employment. As a consequence, our rationale has been to capitalize on the powerful algorithms currently available for spectral comparison. Following this rationale, the first priority is to ensure the generation of MALDI-TOF MS spectra from mixtures of microorganisms that contain manageable peak complexities and that can be handled by the existing spectral comparison algorithms, preferably with the option to archive and re-run sample preparations and to pipette replicates of these onto MALDI-TOF MS sample plates. The second priority is to ensure that database entry is comparably facile to sample preparation so that large databases of known microorganism mixture MALDI-TOF MS spectra could be readily prepared for comparison with the spectra of unknown mixtures. In this article, we address the above priorities and generate illustrative MALDI-TOF MS spectra to demonstrate the utility of this approach. In addition, we investigate methods aimed at chemically modulating the peak complexity of the obtained MALDI-TOF MS spectra.

## Introduction

A microbiome can be defined as a set of genes found within a set of microorganisms (the microbiota) associated with a particular organism or environment. For example, there are some 10–100 trillion microorganisms associated with humans (most of which are bacteria in the gut) [1] and the human microbiome consists of the set of genes found within these microorganisms [2]. A key driver for the gene-based analysis of mixtures of microorganisms has been the National Institutes of Health Human Microbiome Project [3], but other microbiome projects have also been established, increasing in terms of scope up to the Earth Microbiome Project [4]. Since DNA-based microbiome analysis can make certain inferences about microbial taxa associated with a particular organism or environment [5], this enables the investigation of links between changes in taxonomic composition and changes in, for example, human, animal, or plant health [6].

Analytical techniques currently available for the characterization of mixtures of microorganisms are generally based on next-generation sequencing [7], particularly reversible terminator sequencing [8, 9] and nanopore sequencing [10, 11]. These methods are, however, lengthy and fairly expensive (ranging between £1000 and £2500 per run depending on the exact sequencing system used and the costing model employed). Motivated to develop practical and less-expensive methods for characterizing such mixtures, the current article proposes, as an alternative or complement, the use of matrix-assisted laser-desorption and ionization time-of-flight mass spectrometry (MALDI-TOF MS) because, in addition to being both rapid and inexpensive in terms of reagent usage and time required for sample processing [12], this technique is capable of high-resolution discrimination between species [12, 13] as well as even higher resolution discrimination between regional biotypes within species [12–15].

Using MALDI-TOF MS, large proteins can be desorbed intact in the gas phase carrying predominantly a single positive charge [16] by means of the MALDI soft ionization process [17]. Since the time-of-flight of a charged protein along a tube held at high vacuum after acceleration in an electrical field is proportional to the square root of the mass-over-charge ratio for the protein [18], a mass spectrum can readily be generated from the time-of-flight values for such gas phase and charged protein components in a particular biological sample [18]. However, one of the current limitations of MALDI-TOF MS is that characterization and/or identification [18–22] is generally restricted to isolated organisms—axenic cultures in the case of microorganisms [18, 19]. The analytical scope of MALDI-TOF MS could therefore be increased significantly if a method were to be developed whereby the technique could be routinely applied to mixtures of microorganisms.

Taking a MALDI-TOF MS spectrum of a mixture of microorganisms and then comparing the observed spectrum with a large set of modelled composite spectra from individual microorganisms has been bioinformatically demonstrated within a human clinical context [23], but such an approach would likely face a number of impediments under more open conditions. Firstly, the component microorganisms are unlikely to be known in advance for many microorganism mixtures, so the number of candidate spectral mixtures is likely to be enormous. Secondly, many environmental microorganism mixtures are likely to contain microorganisms that cannot be cultured [24], and therefore for which reference MALDI-TOF MS will not be available. Thirdly, patterns of protein expression in microorganisms can vary with culture conditions [25, 26] and so candidate spectra may not be representative of any spectral contribution from the same microorganism within the mixture of interest. Fourthly, the summation of component spectra may not necessarily be linear in the mixture as proteins might compete for desorption and ionization during the MALDI process (indeed, spectral profiles are commonly observed to change even with sample dilution [15]).

Our rationale for the work presented in this article has been to defer the quest for extracting taxonomic composition information from mixtures of microorganisms and instead to capitalize on the exquisite resolving power of MALDI-TOF MS [12–15] coupled with the rapidity, simplicity and low cost of the technique [12] and to exploit as much as possible the powerful algorithms currently available for spectral comparison [27]. Following this rationale, the first priority is to ensure the generation of MALDI-TOF MS spectra from mixtures of microorganisms that contain manageable peak complexities and that can be handled by the existing spectral comparison algorithms, preferably with the option to archive and re-run sample preparations and to pipette replicates of these onto MALDI-TOF MS sample plates. The second priority is to ensure that database entry is comparably facile to sample preparation so that large databases of known microorganism mixture MALDI-TOF MS can readily be prepared for comparison with the spectra of unknown mixtures. In the current article, we address the above priorities and generate illustrative MALDI-TOF MS spectra to evaluate the utility of this approach. In addition, we investigate methods aimed at chemically modulating the peak complexity of the obtained MALDI-TOF MS spectra.

## Materials and methods

### Reagents

The following reagents were purchased from Sigma (Gillingham, UK): ≥99.8% ethanol, ≥ 98% (TLC-grade) α-cyano-4-hydroxycinnamic acid (HCCA) matrix, LC–MS grade acetonitrile, cell-culture grade 1.0 N hydrochloric acid (HCl) and 99% ReagentPlus^®^-grade trifluoroacetic acid (TFA). CHROMASOLV^TM^ LC–MS grade water was purchased from Fluka (Loughborough, UK).

### Bacterial species

For evaluating whether MALDI-TOF MS spectra from mixtures contain manageable peak complexities, and whether these can also be handled by the existing spectral comparison algorithms, we selected six bacterial species, available to us from the CABI Culture Collection [28, 29], and known to be associated with the seeds of rice (*Oryza sativa*) [30–32]: *Pantoea agglomerans* (IMI 347419, ‘A’ on spectral labels—subsequently reclassified on the basis of MALDI-TOF MS analysis as *P. ananatis*), *Curtobacterium citreum* (IMI 359423, ‘B’ on spectral labels), *Stenotrophomonas maltophilia* (IMI 361026, ‘C’ on spectral labels), *Burkholderia glumae* (IMI 364372, ‘D’ on spectral labels), *Rhizobium radiobacter* (IMI 389585, ‘E’ on spectral labels), and *Paenibacillus humilis* (IMI 500835, ‘F’ on spectral labels). All bacterial species were sub-cultured three times on nutrient agar plates to ensure single-colony purity, with monitoring at each stage by direct-transfer MALDI-TOF MS and screening against the Bruker BDAL database of bacterial samples [33] (Bruker, Bremen, Germany).

### Sample preparation

For each bacterial species, biomass was harvested from the final streaked plate using an inoculating loop (taking great care not to remove any agar) and was resuspended in 1 ml of water. This was then vortex-mixed and immediately split into two 500 µl aliquots. Each of these was centrifuged at 14 100 g for 2 min in a miniSpin^®^ plus centrifuge (Eppendorf, Stevenage, UK) and the supernatants were removed. The resulting biomass was then resuspended at 30 mg/ml wet biomass in either Solution 1 [65% (v/v) acetonitrile, 2.5% (v/v) TFA, and 32.5% (v/v) water] or Solution 2 [65% (v/v) acetonitrile and 35% (v/v) 1 M HCl] as indicated, and mixed by vortexing. For the choice of acids, we were guided by an unpublished observation during past MALDI-TOF MS method development, in which fewer peaks were sometimes observed in spectra where the counterions were derived from strong mineral acids such as HCl rather than TFA. TFA- and HCl-based mixtures (in the above Solutions 1 and 2) of bacterial proteins, each equivalent to 5 mg/ml of individual species wet biomass per mixture, were prepared as follows: no protein (negative control), *B. glumae* (+), *C. citreum* (+), *P. ananatis* (+), *P. humilis* (+), *R. radiobacter* (+), *S. maltophilia* (+), *B. glumae* (–), *C. citreum* (–), *P. ananatis* (–), *P. humilis* (–), *R. radiobacter* (–), *S. maltophilia* (–), and All (+), where (+) indicates addition of protein from the species specified and (–) indicates addition of protein from all species *except* the species specified.

For extractions, 10 µl aliquots of the above mixtures were placed in 1.5 ml Eppendorf tubes and 100 µl of Solution 3 [11-mg/ml HCCA matrix, 65% (v/v) acetonitrile, 2.5% (v/v) TFA, and 32.5% (v/v) water] for the TFA-based mixtures or Solution 4 [11-mg/ml HCCA matrix, 65% (v/v) acetonitrile, and 35% (v/v) 1 M HCl] for the HCl-based mixtures were added, followed by vortex-mixing. Triplicate 1 µl aliquots were then pipetted onto the Bruker sample plate, air-dried, and loaded into the spectrometer.

### Mass spectrometry

Mass spectrometry was carried out using a Bruker Microflex LT linear-mode instrument running the MALDI Biotyper 4.0 applications (Bruker Daltonik, Bremen, Germany) as described in [14]. All spectra are shown baseline-subtracted, smoothed, and autoscaled in the Y-direction, covering a range of 2–20 kDa, with X-axis scale increments of 2 kDa. Database entries were made as single-spectra Main Spectra (MSPs) using the Bruker Online Client software suite (version 4.0.19, Bruker Daltonik, Bremen, Germany) using the manufacturer’s standard settings. For spectral comparisons, Bruker identification scores were derived using the standard Bruker algorithm. This first converts raw mass spectra into peak lists, which are then compared between spectra. Three separate values are computed: the number of peaks in the reference spectrum that have a closely matching partner in the test spectrum (value range 0–1), the number of peaks in the test spectrum that have a closely matching partner in the reference spectrum (value range 0–1), and the peak height symmetry of the matching peaks (value range 0–1). The above three values are multiplied together and normalized to 1000, and then the base-10 logarithm is taken to give the final Bruker score (range 0–3). Bruker scores between 2.3 and 3.0 indicate very close relatedness, scores between 2.0 and 2.3 indicate close relatedness, score between 1.7 and 2.0 indicate intermediate relatedness, and scores below 1.7 indicate low relatedness.

### Spectral comparison

Duplicate ‘reference’ sample preparations were carried out as indicated for each mixture of microorganisms, from which a database of reference spectra was generated. For spectral comparison, ‘test’-sample spectra were compared against the database of reference spectra and Bruker identification scores were generated as described above. These were then averaged for each of the reference sample Bruker scores.

## Results

**Figure 1:**
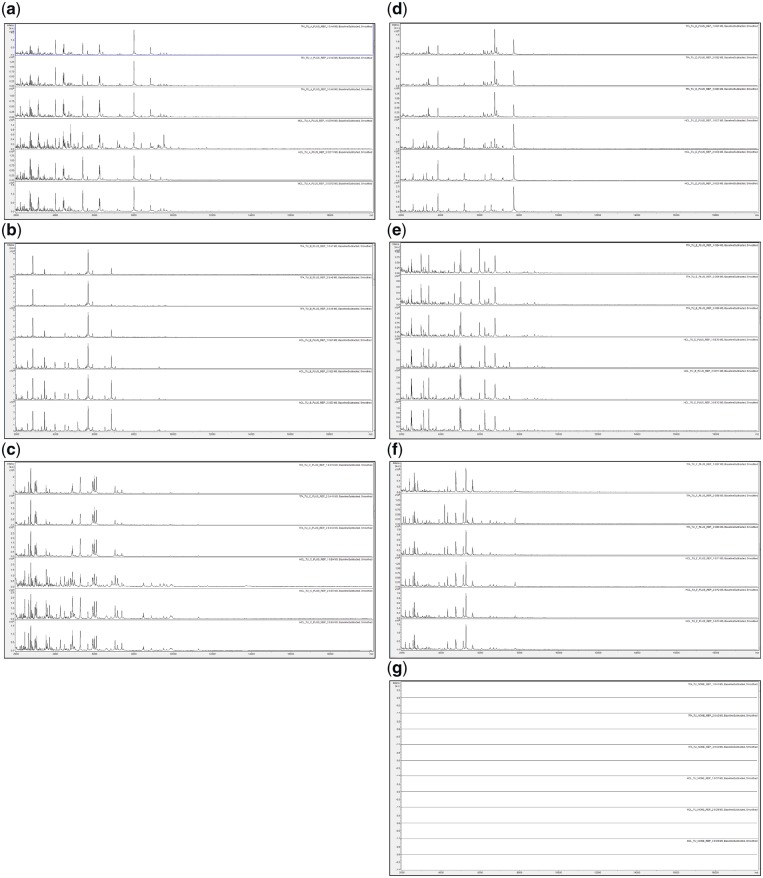
MALDI-TOF MS spectra of acid-soluble proteins for, from top to bottom in each panel, TFA-based extraction (replicates 1–3) and HCl-based extraction (replicates 1–3) for (**a**) *P. ananatis* (+), (**b**) *C. citreum* (+), (**c**) *S. maltophilia* (+), (**d**) *B. glumae* (+), (**e**) *R. radiobacter* (+), (**f**) *P. humilis* (+), and (**g**) negative control.

**Figure 2:**
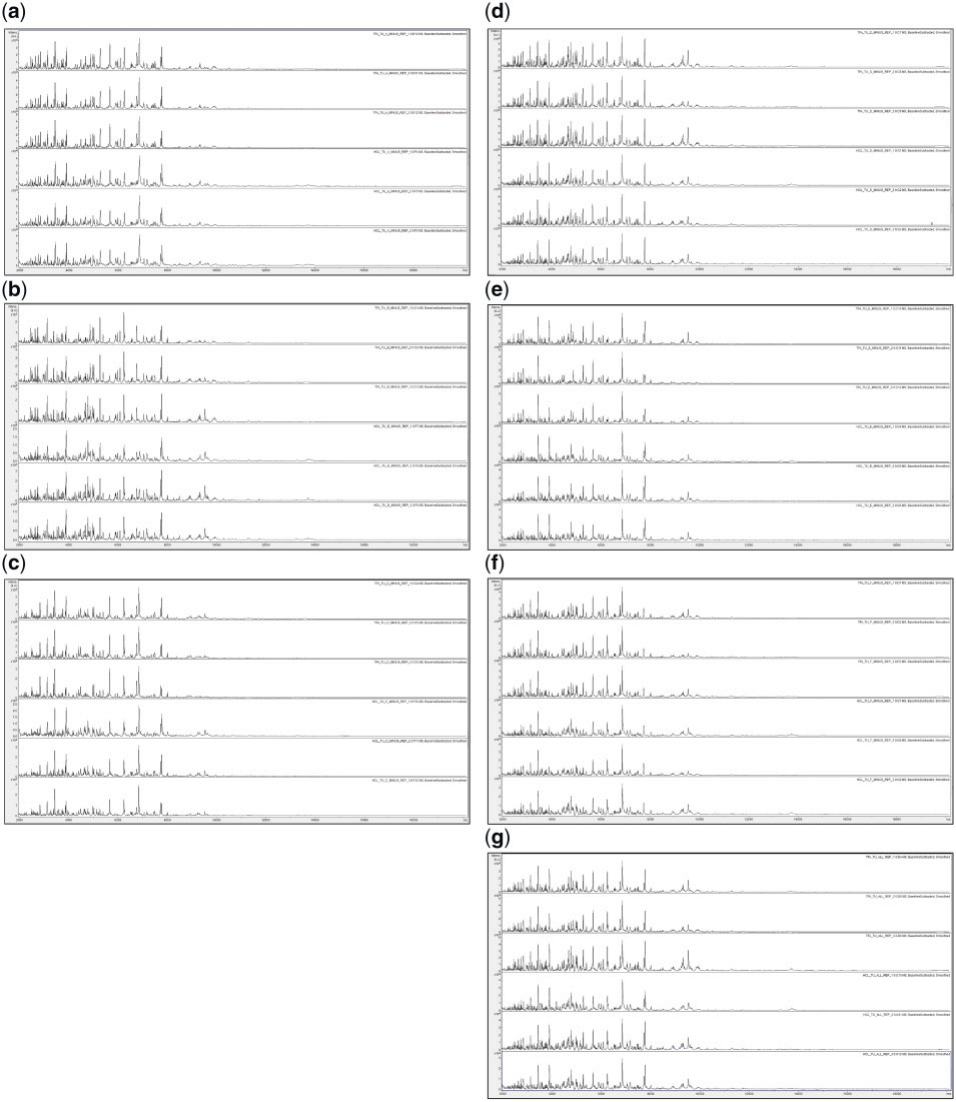
MALDI-TOF MS spectra of acid-soluble proteins for, from top to bottom in each panel, TFA-based extraction (replicates 1–3) and HCl-based extraction (replicates 1–3) for (**a**) *P. ananatis* (–), (**b**) *C. citreum* (–), (**c**) *S. maltophilia* (–), (**d**) *B. glumae* (–), (**e**) *R. radiobacter* (–), (**f**) *P. humilis* (–), and (**g**) All (+).

**Figure 3:**
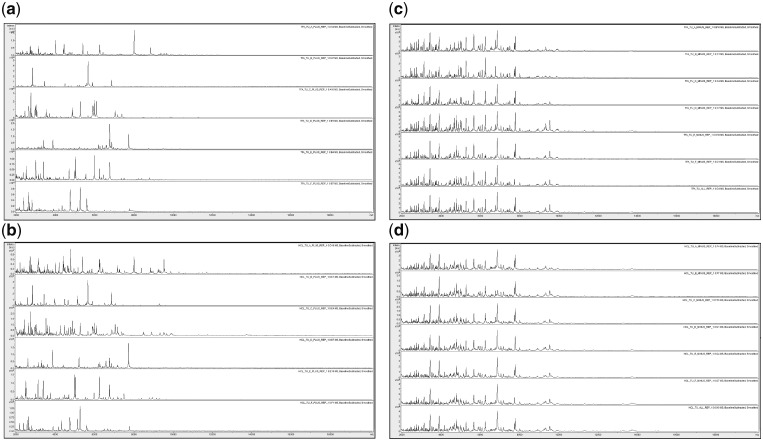
Comparison between replicate-1 MALDI-TOF MS spectra of acid-soluble proteins for, from top to bottom in each panel (**a** and **b**), *P. ananatis* (+), *C. citreum* (+), *S. maltophilia* (+), *B. glumae* (+), *R. radiobacter* (+), and *P. humilis* (+); and, from top to bottom in each panel (**c** and **d**), *P. ananatis* (–), *C. citreum* (–), *S. maltophilia* (–), *B. glumae* (–), *R. radiobacter* (–), *P. humilis* (–), and All (+) for TFA- (**a**–**c**) and HCl-based extractions (**b**–**d**).

Triplicate MALDI-TOF MS spectra of the 28 acid-soluble protein mixtures described in the Materials and Methods section for TFA- and HCl-based extractions are shown in Figs 1–3.

Figure 1 shows good spectral replication, and in two cases (Fig. 1b and c), slightly greater visual peak richness for HCl-based extractions compared with the TFA-based equivalents. Figure 2 again shows good spectral replication, and in some cases (Fig. 2a, c, d, and g), slightly greater visual peak richness for TFA-based extractions. Figure 3 shows visually distinct spectra for the six bacterial species used in this study (but not for the mixtures) using both HCl- and TFA-based extractions.

**Figure 4:**
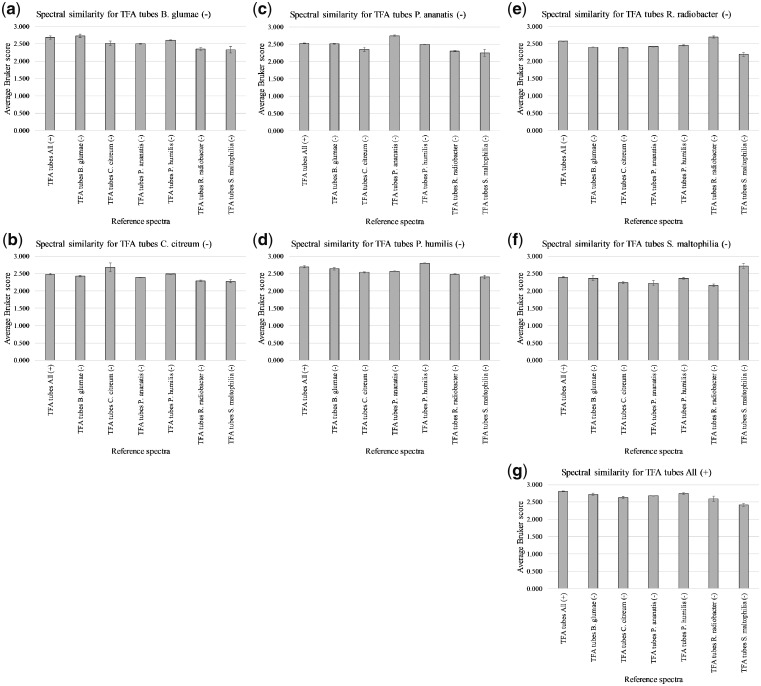
Average Bruker scores for spectral comparison between (–) replicate-1 test samples (**a**) *B. glumae*, (**b**) *C. citreum*, (**c**) *P. ananatis*, (**d**) *P. humilis*, (**e**) *R. radiobacter*, (**f**) *S. maltophilia*, and (**g**) All (+) replicate-1 and (–) replicates 2 and 3/All (+) replicates 2 and 3 reference samples for TFA-based extractions.

Supplementary Table S1 shows the Bruker scores obtained for spectral comparison between the various replicate-1 test samples and replicate 2 and 3 reference samples for TFA-based extractions. These were used to generate average Bruker scores, with error bars indicating 1 standard deviation (SD) on either side of the mean, as shown in Fig. 4.

Figure 4 shows the highest spectral similarity between replicate-1 test samples and their cognate replicate 2 and 3 reference samples in all seven cases. In only one case (Fig. 4a), is the separation too close and the errors too large for clear discrimination. In all seven cases, both cognate average Bruker scores and non-cognate average Bruker scores exceeded 2.0.

**Figure 5:**
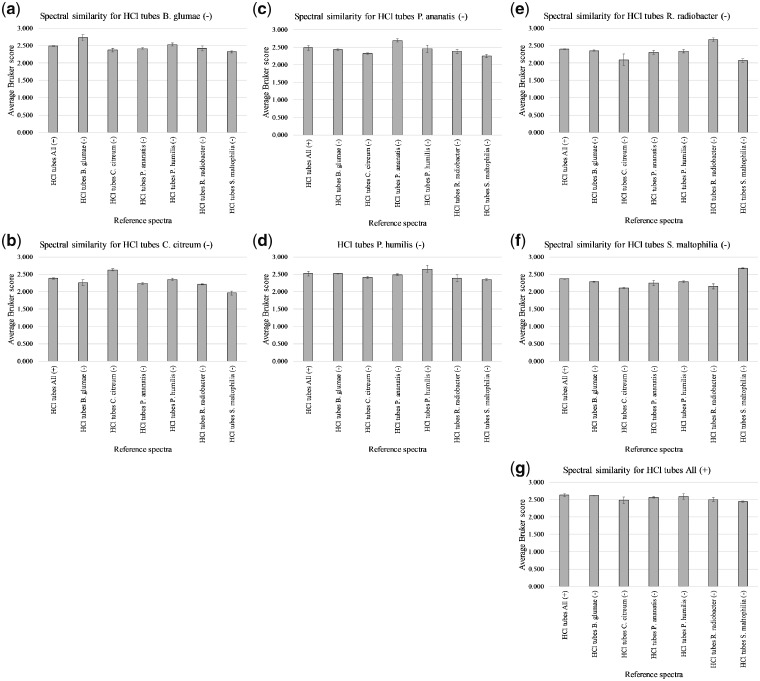
Average Bruker scores for spectral comparison between (–) replicate-1 test samples (**a**) *B. glumae*, (**b**) *C. citreum*, (**c**) *P. ananatis*, (**d**) *P. humilis*, (**e**) *R. radiobacter*, (**f**) *S. maltophilia*, and (**g**) All (+) replicate-1 and (–) replicates 2 and 3/All (+) replicates 2 and 3 reference samples for HCl-based extractions.

Supplementary Table S2 shows the Bruker scores obtained for spectral comparison between the various replicate-1 test samples and replicate 2 and 3 reference samples for HCl-based extractions. These were used to generate average Bruker scores, with error bars indicating 1 SD on either side of the mean, as shown in Fig. 5.

Figure 5 shows the highest spectral similarity between replicate-1 test samples and their cognate replicate 2 and 3 reference samples in all seven cases. In only two cases (Fig. 5d and f), is the separation too close and the errors too large for clear discrimination. In all seven cases, cognate average Bruker scores exceeded 2.0, and in all but one case (Fig. 5b), non-cognate average Bruker scores exceeded 2.0.

## Discussion and conclusions

For the reasons outlined in the Introduction section, rather than trying to infer taxonomic composition information from mixtures of microorganisms, in the current article we have instead chosen to capitalize on the resolving power of MALDI-TOF MS coupled with the powerful algorithms currently available for spectral comparison. In doing so, we have effectively chosen to think of microbial mixtures and ‘one big meta-organism’ and to extrapolate wherever possible from the identification of pure microorganisms. For this approach to bear fruit, the MALDI-TOF MS spectra obtained from mixtures of microorganisms must contain manageable peak complexities that can also be handled by spectral comparison algorithms. The above study clearly shows that manageable peak complexities are obtained from protein mixtures from up to six bacterial species using either TFA- or HCl-based extractions. Moreover, for these, the Bruker spectral comparison algorithm consistently shows the highest spectral similarity between cognate test and reference sample mixtures, and is able to discriminate between many of the very similar protein mixtures tested. In the current article, single-spectra MSPs have been employed for all spectral comparisons so that variation between reference-sample MALDI could be assessed and reported (as 1 SD on either side of the mean above) by making all possible spectral comparisons rather than comparisons between test spectra and multiple-spectra ‘averaged’ MSPs derived from reference-sample replicates. HCl-based extractions were undertaken to investigate whether these could be used to ‘tune’ the peak complexity should the number of peaks observed in the protein mixtures tested prove to be too high. While there is some evidence for slightly reduced peak complexity using HCl, crystal morphology for HCl-based MALDI-TOF MS was observed to be much less uniform than for TFA (with very rapid formation of few large crystals rather than slow formation of many small and fairly uniform crystals), which arguably offsets any marginal advantage in terms of reduced peak complexity.

In addition to generating manageable peak complexities that are compatible with the Bruker spectral comparison algorithm, and in contrast to ‘direct-transfer’ methods [12, 18], our method additionally gives users the option to archive and re-run sample preparations from mixtures of microorganisms and also to pipette replicates of these onto MALDI-TOF MS sample plates, which can then be used to give an average and standard deviation across the replicates used for spectral comparisons. Complementary to the above, database entry for the spectra generated should ideally be of comparable ease and speed to sample preparation so that large databases of known microorganism mixture MALDI-TOF MS spectra could be prepared rapidly and cheaply for comparison with mixture spectra to be tested. To this end, we have again used the ‘single-spectrum MSP’ approach discussed in Reeve and Seehausen [14], which departs slightly from the ‘standard Bruker method’ for routine clinical identifications of bacteria and yeast in that every single spectrum obtained is used to make a separate database entry, a process that takes just a few seconds per database entry.

Technical advantages of the method described above include the fact that a reasonably full picture of the mixture of microorganisms is obtained because spectral contributions from microorganisms that comprise biomass in environmental samples but are not amenable to culture using laboratory media are still observed, and there is also no complication due to polymerase chain reaction (PCR) amplification bias resulting from differences in complementarity between the primer-binding sites and the PCR primers. In addition, a view across multiple expressed genomic loci is obtained rather than analysing the consequences of evolution within a single highly conserved (non-coding) gene. A further advantage may be that, unlike the case for PCR-based methods that do not discriminate between living and dead material, living material ought to be the primary contributor to the observed MALDI-TOF MS spectra from microorganism mixtures (though this will depend on the rate of protein degradation *post mortem* within dead cells, which may not be known in most cases). Economic advantages of the method described above include the fact that sample preparation takes around 30 sec per sample and reagent costs are around 1.2 UK pence per sample [13], which, coupled with the above-mentioned rapid method of database entry, enables the generation of large spectral databases of microorganism mixtures at low cost and with minimal labour input and laboratory infrastructure. Technical disadvantages of the method described above include the fact that it only ‘sees’ molecular weights and their changes that are manifested in the acid-soluble protein fraction, along with the lack of any taxonomic composition information. In addition, unlike the case for PCR-based methods (where the primer sequences confer a high degree of analytical selectivity), host organism material will need to be carefully excluded to prevent this making a contribution to the observed MALDI-TOF MS spectra. For the extraction of acid-soluble proteins from environmental sample biomass, it is also possible that some means of biomass concentration may be needed for some samples. On the above basis, we would contend that the method described above is essentially *complementary* to (rather than competing with) next-generation sequencing, with MALDI-TOF MS serving as a rapid and inexpensive tool for screening samples prior to, if required, more time-consuming and expensive sequencing-based studies and their attendant bioinformatic analysis. Possible applications examples for the method described in this article could include characterization of synthetic microbial consortia for use in plant growth promotion and/or seed treatments and characterization of microbial mixture antigens for the preparation of polyclonal antibodies against such mixtures for use in immunoassays (e.g. those used in fuel-contamination testing)—two areas of interest in which we would like to investigate the validity of our approach.

## Data availability

Original spectral data held on the Bruker Microflex PC is available on request.

## Supplementary Material

bpz013_Supplementary_Data
