# Supplementary material for: MALDI-TOF MS protein fingerprinting of mixed samples
Source: Biol Methods Protoc. 2019 Sep 25;4(1):bpz013. doi: 10.1093/biomethods/bpz013 (PMC7200911; doi:10.1093/biomethods/bpz013)
Supplement: bpz013_Supplementary_Data [file bpz013_supplementary_data.docx]

**Supplementary materials**

**Table S1** Bruker scores for spectral comparison between (-) replicate-1 test samples / (+) All replicate-1 and (-) replicate-2 and -3 / (+) All replicate-2 and -3 reference samples for TFA-based extraction in tubes.

|  | TFA tubes *B. glumae* (-) Rep 1 | TFA tubes *C. citreum* (-) Rep 1 | TFA tubes *P. ananatis* (-) Rep 1 | TFA tubes *P. humilis* (-) Rep 1 | TFA tubes *R. radiobacter* (-) Rep 1 | TFA tubes *S. maltophilia* (-) Rep 1 | TFA tubes All (+) Rep 1 |
| --- | --- | --- | --- | --- | --- | --- | --- |
| TFA tubes All (+) Rep 2 | 2.640 | 2.492 | 2.538 | 2.718 | 2.580 | 2.379 | 2.812 |
| TFA tubes All (+) Rep 3 | 2.718 | 2.464 | 2.516 | 2.674 | 2.578 | 2.404 | 2.801 |
| TFA tubes *B. glumae* (-) Rep 2 | 2.697 | 2.444 | 2.523 | 2.666 | 2.410 | 2.317 | 2.700 |
| TFA tubes *B. glumae* (-) Rep 3 | 2.768 | 2.404 | 2.496 | 2.611 | 2.405 | 2.414 | 2.744 |
| TFA tubes *C. citreum* (-) Rep 2 | 2.556 | 2.762 | 2.392 | 2.550 | 2.374 | 2.251 | 2.613 |
| TFA tubes *C. citreum* (-) Rep 3 | 2.474 | 2.584 | 2.308 | 2.526 | 2.394 | 2.221 | 2.643 |
| TFA tubes *P. ananatis* (-) Rep 2 | 2.493 | 2.393 | 2.735 | 2.566 | 2.427 | 2.170 | 2.682 |
| TFA tubes *P. ananatis* (-) Rep 3 | 2.515 | 2.392 | 2.757 | 2.574 | 2.423 | 2.276 | 2.678 |
| TFA tubes *P. humilis* (-) Rep 2 | 2.616 | 2.481 | 2.488 | 2.790 | 2.446 | 2.379 | 2.724 |
| TFA tubes *P. humilis* (-) Rep 3 | 2.597 | 2.487 | 2.500 | 2.807 | 2.470 | 2.345 | 2.757 |
| TFA tubes *R. radiobacter* (-) Rep 2 | 2.318 | 2.305 | 2.311 | 2.479 | 2.674 | 2.194 | 2.542 |
| TFA tubes *R. radiobacter* (-) Rep 3 | 2.377 | 2.282 | 2.295 | 2.483 | 2.720 | 2.139 | 2.645 |
| TFA tubes *S. maltophilia* (-) Rep 2 | 2.396 | 2.308 | 2.317 | 2.433 | 2.235 | 2.767 | 2.441 |
| TFA tubes *S. maltophilia* (-) Rep 3 | 2.263 | 2.253 | 2.173 | 2.370 | 2.158 | 2.671 | 2.383 |

**Table S2** Bruker scores for spectral comparison between (-) replicate-1 test samples / (+) All replicate-1 and (-) replicate-2 and -3 / (+) All replicate-2 and -3 reference samples for HCl-based extraction in tubes.

|  | HCl tubes *B. glumae* (-) Rep 1 | HCl tubes *C. citreum* (-) Rep 1 | HCl tubes *P. ananatis* (-) Rep 1 | HCl tubes *P. humilis* (-) Rep 1 | HCl tubes *R. radiobacter* (-) Rep 1 | HCl tubes *S. maltophilia* (-) Rep 1 | HCl tubes All (+) Rep 1 |
| --- | --- | --- | --- | --- | --- | --- | --- |
| HCl tubes All (+) Rep 2 | 2.478 | 2.392 | 2.526 | 2.569 | 2.407 | 2.374 | 2.658 |
| HCl tubes All (+) Rep 3 | 2.491 | 2.367 | 2.431 | 2.472 | 2.387 | 2.373 | 2.598 |
| HCl tubes *B. glumae* (-) Rep 2 | 2.665 | 2.202 | 2.450 | 2.525 | 2.342 | 2.296 | 2.620 |
| HCl tubes *B. glumae* (-) Rep 3 | 2.794 | 2.326 | 2.420 | 2.527 | 2.366 | 2.273 | 2.619 |
| HCl tubes *C. citreum* (-) Rep 2 | 2.406 | 2.645 | 2.335 | 2.428 | 2.209 | 2.085 | 2.547 |
| HCl tubes *C. citreum* (-) Rep 3 | 2.330 | 2.602 | 2.302 | 2.381 | 1.970 | 2.104 | 2.410 |
| HCl tubes *P. ananatis* (-) Rep 2 | 2.428 | 2.214 | 2.660 | 2.467 | 2.260 | 2.198 | 2.548 |
| HCl tubes *P. ananatis* (-) Rep 3 | 2.398 | 2.251 | 2.730 | 2.506 | 2.341 | 2.305 | 2.579 |
| HCl tubes *P. humilis* (-) Rep 2 | 2.498 | 2.372 | 2.380 | 2.575 | 2.297 | 2.301 | 2.533 |
| HCl tubes *P. humilis* (-) Rep 3 | 2.558 | 2.327 | 2.531 | 2.730 | 2.373 | 2.266 | 2.646 |
| HCl tubes *R. radiobacter* (-) Rep 2 | 2.369 | 2.203 | 2.345 | 2.313 | 2.632 | 2.098 | 2.466 |
| HCl tubes *R. radiobacter* (-) Rep 3 | 2.467 | 2.225 | 2.416 | 2.452 | 2.702 | 2.199 | 2.539 |
| HCl tubes *S. maltophilia* (-) Rep 2 | 2.344 | 1.918 | 2.286 | 2.332 | 2.032 | 2.663 | 2.460 |
| HCl tubes *S. maltophilia* (-) Rep 3 | 2.299 | 1.997 | 2.215 | 2.368 | 2.112 | 2.678 | 2.434 |
